# Earthquake-Induced Building-Damage Mapping Using Explainable AI (XAI)

**DOI:** 10.3390/s21134489

**Published:** 2021-06-30

**Authors:** Sahar S. Matin, Biswajeet Pradhan

**Affiliations:** 1 Centre for Advanced Modelling and Geospatial Information Systems (CAMGIS), Faculty of Engineering and IT, University of Technology Sydney, Ultimo, NSW 2007, Australia; sahar.soleimanimatin@student.uts.edu.au; 2 Department of Energy and Mineral Resources Engineering, Sejong University, Choongmu-gwan, 209 Neungdong-ro, Gwangjin-gu, Seoul 05006, Korea; 3 Center of Excellence for Climate Change Research, King Abdulaziz University, P.O. Box 80234, Jeddah 21589, Saudi Arabia; 4 Earth Observation Center, Institute of Climate Change, Universiti Kebangsaan Malaysia, Bangi 43600, Selangor, Malaysia

**Keywords:** building-damage mapping, feature analysis, explainable AI, machine learning, remote sensing

## Abstract

Building-damage mapping using remote sensing images plays a critical role in providing quick and accurate information for the first responders after major earthquakes. In recent years, there has been an increasing interest in generating post-earthquake building-damage maps automatically using different artificial intelligence (AI)-based frameworks. These frameworks in this domain are promising, yet not reliable for several reasons, including but not limited to the site-specific design of the methods, the lack of transparency in the AI-model, the lack of quality in the labelled image, and the use of irrelevant descriptor features in building the AI-model. Using explainable AI (XAI) can lead us to gain insight into identifying these limitations and therefore, to modify the training dataset and the model accordingly. This paper proposes the use of SHAP (Shapley additive explanation) to interpret the outputs of a multilayer perceptron (MLP)—a machine learning model—and analyse the impact of each feature descriptor included in the model for building-damage assessment to examine the reliability of the model. In this study, a post-event satellite image from the 2018 Palu earthquake was used. The results show that MLP can classify the collapsed and non-collapsed buildings with an overall accuracy of 84% after removing the redundant features. Further, spectral features are found to be more important than texture features in distinguishing the collapsed and non-collapsed buildings. Finally, we argue that constructing an explainable model would help to understand the model’s decision to classify the buildings as collapsed and non-collapsed and open avenues to build a transferable AI model.

## 1. Introduction

Using remote-sensing imagery for damage mapping dates back to the San Francisco earthquake in 1906, when a number of kites were used as aerial platforms to capture images from the affected area [1]. A preliminary attempt to use satellite images in the seismic field was probably in the study conducted in 1972 to investigate the cause of the 1964 Alaska earthquake [2]. This makes post-earthquake damage mapping one of the oldest applications of remote-sensing images. However, this topic is challenging in terms of automatic damage assessment, and therefore, it is still the subject of active research [3,4,5,6].

Remote-sensing-based damage mapping is limited to the visual interpretation of optical satellite images in real-world scenarios, which is labour-intensive and time-consuming [7,8]. Therefore, there is an interest in automatic post-earthquake damage mapping. Artificial intelligence (AI) has been widely used in automatic damage-mapping studies using remote-sensing images [9,10,11]. However, AI-based solutions have not been yet entirely adapted to earthquake-induced damage mapping [12]. One reason that hinders AI’s reliable use in real-world applications is the lack of explainability of AI models to justify the AI decisions [13].

Recently, explainability has become extremely crucial for the practical deployment of AI models, and there is a growing interest in this domain [14]. The explainability of the AI model is even more important in the domains, such as post-earthquake rapid damage-mapping, that have a high level of resistance to error. It means that any error in detecting collapsed buildings may cause a delay in rescuing the trapped victims and consequently, can affect human life [9]. Exploring how the AI-model inputs impact the output leads us to better understand the behavior of the model and aids us in building a more robust explainable AI model [15]. Explainable AI (XAI) refers to the AI models of which the output can be understood by humans, which is the opposite of the term “black box” for machine learning models. Therefore, XAI models provide insight into the causes of the model output [16]. Further, the explainability can help identify the unknown vulnerabilities and flaws of the AI system and therefore, enable us to correct errors [15].

The foundation of any machine learning model is the training dataset which is difficult to collect in the building-damage assessment domain as the number of collapsed buildings is usually far fewer than non-collapsed buildings, and there is a high diversity in the type of damages to the buildings [17]. The training dataset in this domain needs to include annotated image samples of the affected area. Further, the annotation should follow a standard damage class guideline [18]. Then the relevant features should be extracted from the annotated image samples to be fed into an ML model. An explainable AI algorithm can then be employed to interpret the model and to explain the impact of the features on the model output.

Therefore, this study emphasises explaining a building-damage-detection model output which leads us towards an understanding of: (1) how/which features contribute to the model output; (2) how the dataset characteristics affect the model output, and (3) how features behave differently in geographically diverse study areas. The insight we gain from using XAI for building-damage-detection models can be used as a rationality check for the training dataset, which is fundamental to machine learning models. To the best of our knowledge, there is no study examining the earthquake-induced building-damage assessment models regarding their explainability, to enhance the model and to identify existing limitations in the dataset, which is essential for building a transferable model.

## 2. Related Work

There have been a significant number of studies proposing different automatic and semi-automatic frameworks using a variety of image types (optical, synthetic aperture radar (SAR) and light detection and ranging (LiDAR)) for earthquake-damage assessment [3,19]. To understand which features contribute to detecting damaged buildings, researchers examined the use of different types of features and the combination of them in assessing damage after an earthquake. These features vary across different studies in the literature, depending on input image type, the use of multisource or multiresolution images, the availability of the ancillary data (e.g., digital elevation model (DEM)), and the capability of the method to handle a multidimensional dataset. Apart from the type of the features, the number of features is also different across the studies and ranges from four [9] to about forty [20].

One of the popular approaches in assessing damage to buildings is calculating the changes in features extracted from a pair of images captured before and after the earthquake, which is called change detection [18]. These features include, but are not limited to, spectral, textural, edge, spatial relationship, and geometry information [20]. After calculating the value of the changes in feature values, a threshold is set by the experts or according to the ground truth to identify the meaningful changes and hence, estimate the damaged area. Taking this approach, researchers (Table 1) used different features to detect damaged or collapsed buildings. For example, Gamba et al. [21] proposed an automatic change-detection method that could detect about 70% of collapsed buildings based on shape analysis. Yusuf et al. [22] compared the brightness value of pre- and post-earthquake Landsat-7 images to detect damaged areas. Ishii et al. [23] considered differences in the colour of a pair of co-registered images to detect the damaged area. Zhang et al. [24] used the mean and variance of the intensity of greyscale pre- and post-event images to detect the damage grade of the building blocks. Rathje et al. [25] used a combination of texture and a correlation coefficient to detect changes after an earthquake, whereas Pesaresi et al. [26] used radiometric and morphological features to detect collapsed buildings. Turker et al. [27] used the temporal DEMs generated from the pre- and post-event aerial images to detect collapsed buildings. Rezaian et al. [28] and Rezaian [29] used a combination of digital surface model (DSM), pixel density, and segment shape to identify the level of damage to buildings.

Studies show that using information obtained from SAR or LiDAR images combined with features extracted from optical images can improve damage assessment [30,31]. Height information extracted from LiDAR images and spectral and textural features from optical post-event images has been used by Ural et al. [32] and Yu et al. [33].

As a cloud-free pre-event optical image might not always available, using change-detection techniques is limited. Therefore, with the emergence of very high-resolution remote-sensing images, such as Worldview 3 and TerraSAR-X, extracting the relevant features using only post-event images is an alternative solution. However, a few studies used only post-earthquake satellite images from an urban complex scene to assess the damages to the buildings. Some of these studies have been summarised in Table 1.

**Table 1:** Studies that have used different feature descriptors, methods, and image types.

Main Findings	Features	References
Detected the collapsed buildings	Shape	Gamba et al. [21]
	Radiometric and morphological	Pesaresi et al. [26]
	DSM	Turker et al. [27]
	Radiometric and morphological	Pesaresi et al. [26]
	Colour, shape, texture, height	Yu et al. [33]
Classified buildings as damaged and non-damaged	Spectral, textural, and spatial relations	Li et al. [34]
	Variance and the direction of edge intensity, texture	Mitomi et al. [35]
	Spectral, textural, and structural	Cooner et al. [36]
	Textural and colour	Rasika et al. [37]
	Spectral and textural	Rathjeh et al. [25]
	Spectral, textural, and height	Ural et al. [32]
	geometric	Wang et al. [38]
Classified buildings as damaged, destroyed, possibly damaged, or no damage	Spectral, intensity, and coherence, DEM	Adriano et al. [20]
Classified the image into debris, non-debris, or no change	Spectral, textural, and brightness, shape	Khodaverdizahraee et al. [39]
Classified buildings as collapsed, partially collapsed, or undamaged	Normalised DSM, pixel intensity, and segment shape	Rezaian et al. [28] Rezaian [29]
Classified buildings as non-damaged, slightly damaged, low level of damage,	SAR texture	DellAqcua et al. [19]
Identified damaged areas	Spectral	Syifa et al. [9]

Considering the variety of image features used in assessing damage to buildings, it is crucial to understand what features contribute to the detection of damage. Identifying the important features to involve in analysis has long been in researchers’ interest. For instance, to understand which feature leads to the best damage-assessment result, Tomowski et al. [40] compared the overall accuracy of four change-detection scenarios using different texture features (contrast, correlation, inverse distance moment, and energy) and concluded that utilising principal component analysis (PCA) with energy achieves the best result. However, using PCA to reduce the dimensionality eliminates the interpretability of the features, as PCA features are the combination of actual features and hence, are not interpretable.

Mansouri and Hamednia [41] employed a genetic algorithm (GA) optimiser to select the optimum features among eight texture features to classify building damage into three classes of no, slight, moderate, and heavy destruction. Three features, “mean”, “dissimilarity” and “second moment”, were selected by the GA optimiser and subsequently used the support vector machine (SVM) classification method. In an attempt to select the most impactful features that contributed to distinguishing the changed and unchanged areas, Khodaverdizahraee et al. [39] utilised the GA optimiser. All of the features were extracted from a pair of optical satellite images captured before and after the Bam earthquake. The initial features included 29 texture and spectral features, from which only nine features were selected after performing GA. However, performing a genetic algorithm is computationally expensive, as the model should be trained for each feature [42].

Employing multisource multiresolution images can slightly increase building-damage-detection accuracy. However, exploring the effect of different datasets and the related features involved in the analysis becomes crucial when the dataset dimension increases due to utilising data fusion. Adriano et al. [20] designed a comparison study in which different scenarios were considered, to compare classification results in the presence or absence of different datasets. A total of eight different scenarios were defined using different combinations of pre and post-event datasets. In their study, the dataset included 11 images (five pre-event images and six post-event images) acquired from different sensors, including ALOS-2 PALSAR-2, Sentinel-1, Sentinel-2, and Planet Scope. The DEM and the building-footprint data were considered ancillary data to contribute to generating the damage map. Three machine learning classifiers, including random forest (RF), rotation forest (RoF), and canonical correlation forest (CCF) were used for pixel-based classification and to generate a damage map. The results from this study showed improvement in accuracy as more datasets from both SAR and optical images were added to the learning process. As a result of this change, the maximum accuracy was achieved for all three classifiers in the scenario in which all the datasets were utilised. Regarding the importance of image features in classification, the result from computing variable importance measurement (VIM) indicated that coherence information derived from multitemporal SAR images contributed slightly more than optically derived features. Further, the VIM results showed that DEM was found to be the most important feature used in the classification model. Although DEM can be used in calculating an earthquake risk factor for specific areas with significant variation in elevation in tsunami-prone areas [43], it cannot be generally transferred to all geographic areas.

Although using both pre-and post-event images increases classification accuracy, the use of only post-event images avoids pre-processing work, like co-registration and histogram matching [39,44]. Moreover, remote-sensing images contain a huge amount of information that can be extracted and used for different purposes. Including a large number of features leads to a large-dimension dataset. Handling such a dataset is time-consuming and computationally expensive. Furthermore, redundant features may add a layer of complexity to the model and thus, decrease the accuracy [45]. Therefore, identifying the optimum features, as well as removing the redundant ones, is a necessary pre-processing step to decrease the dimensionality of the data without a decrease in model performance [46].

Therefore, the main objective of this study is to address the limitation described above and construct an explainable model by (i) using a single post-event optical image for detecting collapsed buildings, (ii) selecting the best image features based on the model output, and (iii) investigating the performance of multilayer perceptron (MLP) in distinguishing collapsed and non-collapsed buildings.

## 3. Method

**Figure 1:**
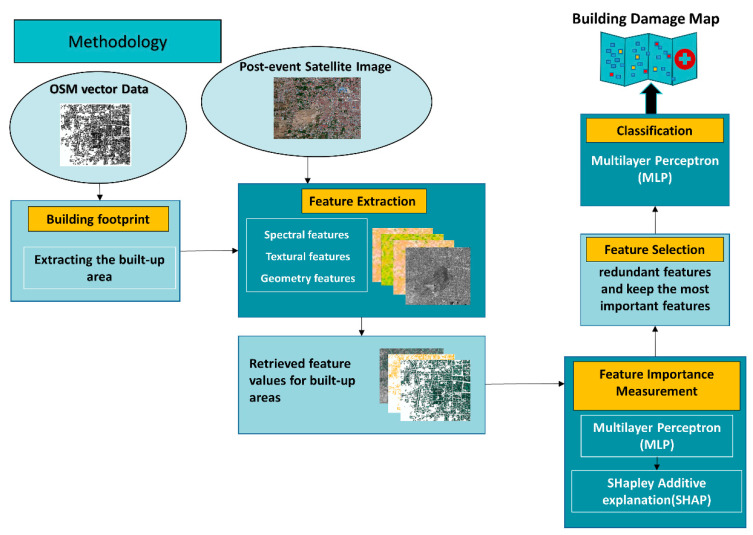
Flowchart of the proposed method for generating a building-damage map.

An optical satellite image captured after the 2018 Palu/Indonesia earthquake was used in this study. Further details about the study area and data can be found in Section 4. The overall methodology of the proposed framework is shown in Figure 1. As shown in this figure, in the first step, different image features (i.e., textural, spectral, and shape features) were extracted from the post-earthquake satellite image. Open street map (OSM) vector data of the affected region was used for the building footprint and then the features’ value was retrieved for each building in the built-up area. Five different built-up areas were considered as the test dataset, and the rest of the buildings were used for training the multilayer perceptron (MLP) model. The Shapley additive explanation (SHAP) algorithm was used for ranking the most impactful features on the model output. Considering the SHAP results, the least impactful features (i.e., the lowest-ranked features) were removed from the dataset to examine the performance of the model after removing these features. Next, the remaining features were included in the model to classify collapsed and non-collapsed buildings. The final building-damage map was generated in two classes, collapsed and non-collapsed. Further details of the method are described in the following subsections.

### 3.1. Multilayer Perceptron

Artificial neural networks are an algorithm that attempts to emulate the structure of the biological neurons in the brain. The multilayer perceptron is an example of an artificial neural network that has been used extensively in image classification [47].

**Figure 2:**
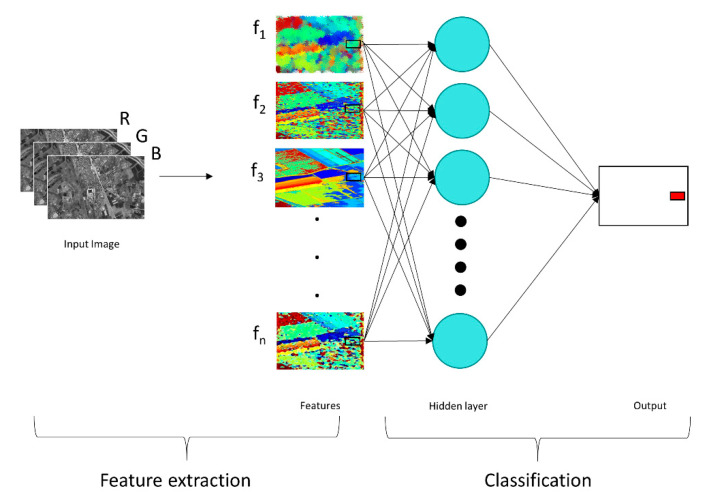
Structure of MLP for building-damage classification (the images/features in this figure are symbolic and are not the real images/features used in this study).

The architecture of the multilayer perceptron (MLP) consists of three main elements, input, hidden layers and output; the inputs are the image features. Each node in MLP is a neuron with non-linear activation function [48]. MLPs utilise a supervised learning technique called backpropagation for training. MLPs have a fully connected structure wherein each perceptron is connected to all other perceptrons (Figure 2).

MLPs utilise a backpropagation algorithm to update the network weights for a training dataset [49]. In each epoch, the network-predicted output is compared to the actual output and the calculated error is propagated back to the networks and therefore, the weights are updated accordingly to minimise the error. Table 2 shows the hyperparameters used in the proposed model.

### 3.2. Feature Extraction

The fundamental step of image classification is selecting the meaningful features that describe the information that can be extracted from an individual or a group of pixels in an image [50]. In this study, spectral and textural features were calculated for each pixel and then averaged for the group of pixels within the building polygon boundary (i.e., image objects) within each building boundary. To avoid over- and under-segmentation and considering the multiscale nature of geographical objects in the image, the non-homogeneous materials used in the building roofs, and the large uniform region in the liquefaction area, we integrated the building vector data in the segmentation process. The details of this method can be found in the study by Kato et al. [51].

Texture features are referred to as the spatial distribution of the intensity values in the image [52]. These features can represent the smoothness or roughness of a group of pixels in the image. Texture features can be calculated for each pixel and have been widely used in the domain of building-damage detection [33,39]. Texture features can be extracted using several texture-analysis methods, such as a Gabor filter, fractal dimension and Haralick. Among these, the grey-level co-occurrence matrix (GLCM) and the grey-level difference vector (GLDV) have been reported to perform well for building-damage detection [4,33,39]. GLCM is a matrix of grey-level distributions for a pair of pixels at a specific distance and orientation, while GLDV counts the number of instances of a given difference between pixels. These features contain information about the position of the pixels with the same grey-level values. Therefore, two sets of GLCM and GLDV texture statistical features were calculated for each pixel in this study. The features were calculated over all the shifts of 1 × 1 of 0, 45, 90, and 135 degrees, using a 3 × 3 pixel window, and 32 as the number of greylevel for each pixel in the image. Then the average of these statistics was returned for each pixel. These features and their corresponding mathematical expressions are shown in Table 3, where $P_{\left(i,j\right)}$ is the normalised value of the pixel *i*,*j*; $V_{K}$ is the image object level, and *N* is the number of rows and columns in the image object.

Spectral features represent the mean intensity value variation in different image bands. These features are used to describe different image objects based on their spectral information [50]. The spectral features used in this study and their relative mathematical expressions are shown in Table 4, where ${\bar{C}}_{i}\left(v\right)$ is the mean intensity of image band *i* for object v; $P_{v}$ is the set of pixels in that object, and #$P_{v}$ is the number of pixels in the set. $w_{k}^{B}$ is the brightness weight of image band *k*; $c_{k}\left(x,y\right)$ is the intensity value at the pixel (x,y), and $B_{v}\left(d\right)$ is the bounding box around the object v with the distance d.

Several image-object shape features have been used in many post-earthquake building-damage-detection studies [33,38,39]. Three different shape features were extracted in this study. Density describes the distribution of the pixels in the object image [52]. Asymmetry is the ratio of length and width of the image objects, and rectangular fit indicates how well the object can fit into a rectangle. The selected shape features are shown in Table 5, where $\rho_{v}\left(x,y\right)\;$ the elliptic distance at a pixel is  (x,y); Var X and Var Y are the variance of X and Y,  respectively.

The values of these features was normalised before feeding them into the MLP classifier. However, they need to be denormalised later to analyse the results using SHAP plots.

### 3.3. Feature Analysis

Remote-sensing images contain a huge amount of information that can be extracted and used for building-damage detection [46]. However, the inclusion of more features in any machine-learning-based classification model may not yield a better result. More features could lead to the presence of redundant or irrelevant features, adding a layer of complexity to the model, thereby increasing the computing time and maybe even leading to a decrease in accuracy [45]. By selecting the most relevant features to distinguish between collapsed and intact buildings, there is a higher likelihood of achieving a good result even without using an advanced machine learning model and hyper-parameter tuning. Therefore, feature selection is a critical pre-processing step in classification studies [53].

This stage aims to explore the impact of the extracted features on the model output and therefore, remove the redundant features and select the best features to include in the modeling. To this end, SHAP summary plot has been used to rank the impact of the features on the capability of the model to distinguish collapsed and non-collapsed buildings.

### 3.4. Shapley Additive Explanations (SHAP)

The Shapley additive explanations value (SHAP) was first introduced for calculating the contribution of the individual players in a coalition game [54]. Recently, Lundberg et al. [55] raised this concept as the basis for an algorithm measuring the importance of the features in machine learning models to explain the output of any ML model. In this case, reproducing the model output considered as the coalition game, in which the players are all the features used in the model, and the number of the observations (number of samples) is the number of times the game is played.

The Shapley value provides the global interpretability of the model by estimating the general contribution of each feature on the model output, as well as producing local interpretability by estimating it for each observation. The Shapley value is calculated based on the average of the marginal contribution across all of the possible permutations of the features. The mathematical expression of the classical SHAP value is given in Equation (1).

(1) $$\emptyset_{i}=\sum_{S\subseteq N\backslash \left\{i\right\}}\frac{\left|S\right|!\left(n-\left|S\right|-1\right)!}{n!}\left[v\left(S\cup \left\{i\right\}\right)-v\left(S\right)\right]$$

where, $\emptyset_{i}$ is the contribution of feature *i*; *N* is the set of all the features; *n* is the number of features in *N*; *N*\{*i*} is the set of all features except *i*; S is any subset of *N* not containing the feature *i*, and v(N) is the base value meaning the predicted output for each feature in *N* without knowing the feature values.

The model output for each observation is estimated by summing up the SHAP value of each feature for that observation. Therefore, the explanation model is formulated as below:

(2) $$g\left(z^{\prime }\right)=\emptyset_{0}+\sum_{i=1}^{M}\emptyset_{i}z_{i}^{\prime }$$

where $z^{\prime }\;\epsilon \;{\left\{0,1\right\}}^{M}$, and *M* is the number of features. $\emptyset_{i}$ can be obtained from Equation (1). As shown in Equation (2), summing up the effect of each feature (SHAP value) approximates the output of the model. SHAP provides multiple AI model explainers. Details about the different model explainers are beyond the scope of this study. The readers are referred to Molnar [56]. Using the relevant model explainer, the global and local importance of the features can be achieved using the summary plot and the force plot, respectively.

#### 3.4.1. SHAP Summary Plot

The summary plot depicts both the feature importance ranking and the feature effects. The term feature effect describes how each feature contributes to the classification output (the *X*-axis on the SHAP summary plot), while the feature importance describes how each feature contribute to the classification performance (the *Y*-axis on SHAP summary plot). Each point in the summary plot represents the Shapley value for a feature and an observation in the dataset. The position of the feature on the *Y*-axis determines the importance of that feature, and the *X*-axis represents the Shapley value of each observation. The colour on the right of the plot shows the value of each observation from low to high; hence, the direction of the relationship between the feature and prediction is shown.

#### 3.4.2. The Individual Force-Plot

Apart from the global interpretability, SHAP provides local interpretability by enabling us to identify the impact of each feature on classifying the individual objects in the image. This capability allows us to analyse the correctly classified and misclassified objects in more detail, which leads us to a deeper understanding of the dataset and the model.

## 4. Experiment and Results

### 4.1. Study Area

Sulawesi Island is located at the convergence of the Indian-Australian Plate, the Pacific Plate, and the Eurasian Plate, and there are several active faults in this region, making this area one of the most vulnerable regions to earthquake. Jena et al. [43] studied the largest earthquake in this region, which happened in 1996 (7.9 Mw), affecting about 100 ${\mathrm{km}}^{2}$ of Sulawesi Island.

On 28 September 2018, a strong earthquake (7.4 Mw) hit Central Sulawesi in Indonesia. As a result of this massive earthquake, 2081 casualties and 68,451 damaged houses were reported [57]. A large area of Palu Bay in this region was affected by the resultant tsunami and, as a result of the intense shaking, liquefaction occurred in the southwest of the bay [9].

**Figure 3:**
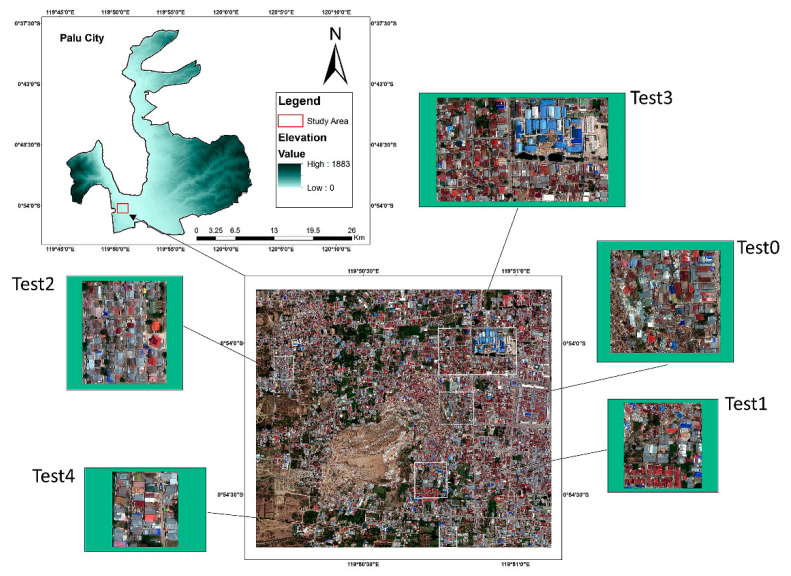
Study area including the training and test areas.

By using remote-sensing images, several emergency management agencies began to publish a rapid damage map to support the rescue and relief activities in this region. For instance, the Copernicus Emergency Management Service was activated on the day of the event and soon published a satellite-based map of Palu City showing a preliminary assessment of damage to buildings and roads [58]. International Charter also was activated a day after the event and provided several damage-assessment maps by comparing pre-event and post-event images of the region [59]. In this research, in order to conduct an earthquake-induced building-damage assessment, part of the affected region in the southwest of Palu Bay was considered as the study area. Figure 3 shows the location of the study area.

Table 6 summarises different datasets employed to prepare the dataset used in this study.

### 4.2. Data

**Figure 4:**
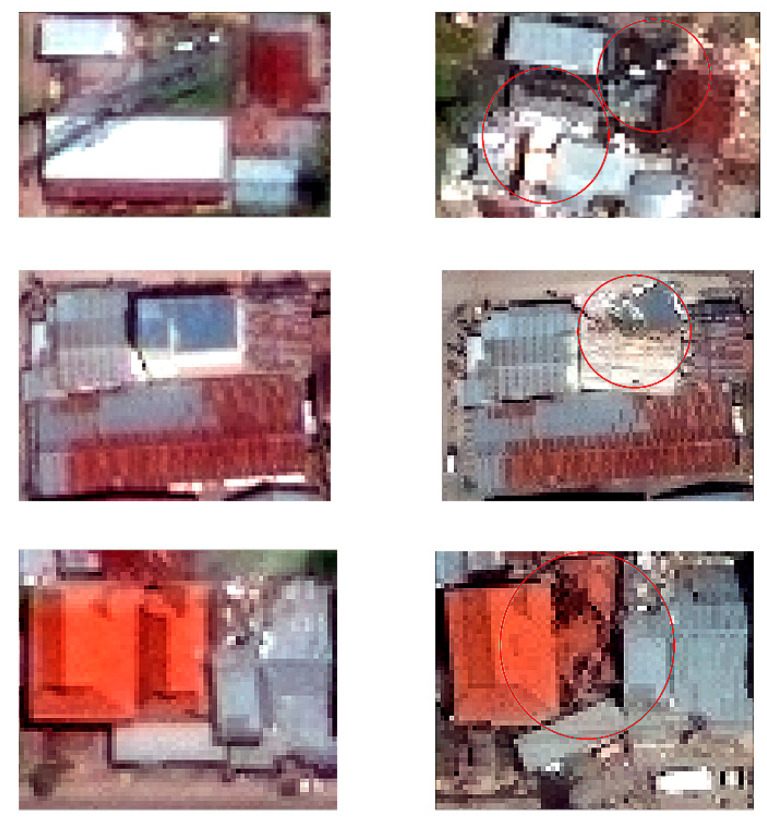
Samples of the pre-event buildings and the corresponding post-event buildings.

The DigitalGlobe open data programme aims to provide high-resolution satellite-imagery products to support rescue and relief activities in affected regions after major natural or human-made hazards [60]. The pre-and post-event satellite images for affected areas are available in the DigitalGlobe open data programme (Figure 4). However, due to the complex distortion of the ground surface after the 2018 massive earthquake, the co-registration of the images can be erroneous in some parts of the study area [44]. Therefore, in this study, only a post-event image was considered for analysis. The WorldView-3 satellite image captured on 2 October 2018 was acquired from the DigitalGlobe open data programme for this study.

From this area, an area of 3606 × 3148 pixels, equal to about 3${\;\mathrm{km}}^{2}$ in the southwest of Palu Bay was selected as the study area. The built-up area in the selected region was about 2821 m^2^. Due to the severity of the earthquake, ground distortion, and the liquefaction in the region, the number of collapsed buildings was significant in this study area [44]. However, the majority of the collapsed buildings were located in the middle of the study area. Therefore, in order to explore the performance of the model in the test areas with different proportions of collapsed and non-collapsed buildings, five test areas were considered, as shown in Figure 3.

### 4.3. Building Footprint

With the increase in geo-information services and the effort of digital volunteers in collaborative projects such as missing maps [61], MapSwipe App [62], and Open Street Map [63] building-footprint data will be more in developing countries [64]. In this study, we utilised the Open Street Map (OSM) building-footprint data. It is important to mention that the quality of the OSM vector data may vary for different areas and in the absence of a building layer, there are land-cover classification techniques that can be used for extracting built-up areas from Earth observation data [65,66]. Open Street Map (OSM) provides geospatial data across the world; thus, the geocoded editable map is available for the public. In this study, we created an area of interest to download the OSM built-up area layer in the region [63].

Due to the distortion of the ground after the earthquake, co-registration of the polygons and the optical image captured by Digital Globe was challenging and hence, some of the polygons were manually shifted to align the buildings and the corresponding polygon in the image [44].

### 4.4. Copernicus Emergency Management Services

The Copernicus Emergency Management Service (EMS) is part of the European Union’s Earth Observation programme that provides different mapping products, including but not limited to, natural-hazard rapid maps and digital geoinformation on a global scale [67].

Copernicus EMS published a grading rapid-damage map along with a building-damage label dataset, in a point format, for the earthquake in Indonesia on 2 October 2018 [58]. The dataset shows different levels of damage to buildings (i.e., destroyed, damaged, possibly damaged and no visible damage). The number of buildings in each class is very different for the region of interest, and hence, the extracted dataset is highly skewed. Further, the low-resolution of the images do not allow us to detect the degree of damage to buildings. Therefore, we considered the damaged, possibly-damaged, and no-visible-damage building classes as non-collapsed and limited the study to classifying them into collapsed and non-collapsed buildings. Some of the damaged buildings in the region of interest were not assigned by a label, as some label points do not correspond to the buildings after co-registration of the dataset and the image. Therefore, we manually labeled the remaining buildings based on the Copernicus EMS damage-assessment classes (Table 7). The overview of the quantity of collapsed and non-collapsed buildings in the training and test datasets is shown in Table 8. As mentioned above, the selected test areas are diverse in terms of containing different portions of collapsed and non-collapsed buildings. Further, due to the contiguous built-up area, cropping a test area that covers the complete buildings in the border of the test area is very challenging; hence, this difficulty led us to selecting areas with a limited number of buildings, such as in test 2 and test 4. 

## 5. Discussions

In the pre-processing step, the post-event satellite images acquired from the Digital Globe open data programme and OSM polygon dataset were co-registered. The built-up area included 8508 buildings, in which 2135 buildings were labelled as collapsed and the rest were considered non-collapsed.

**Figure 5:**
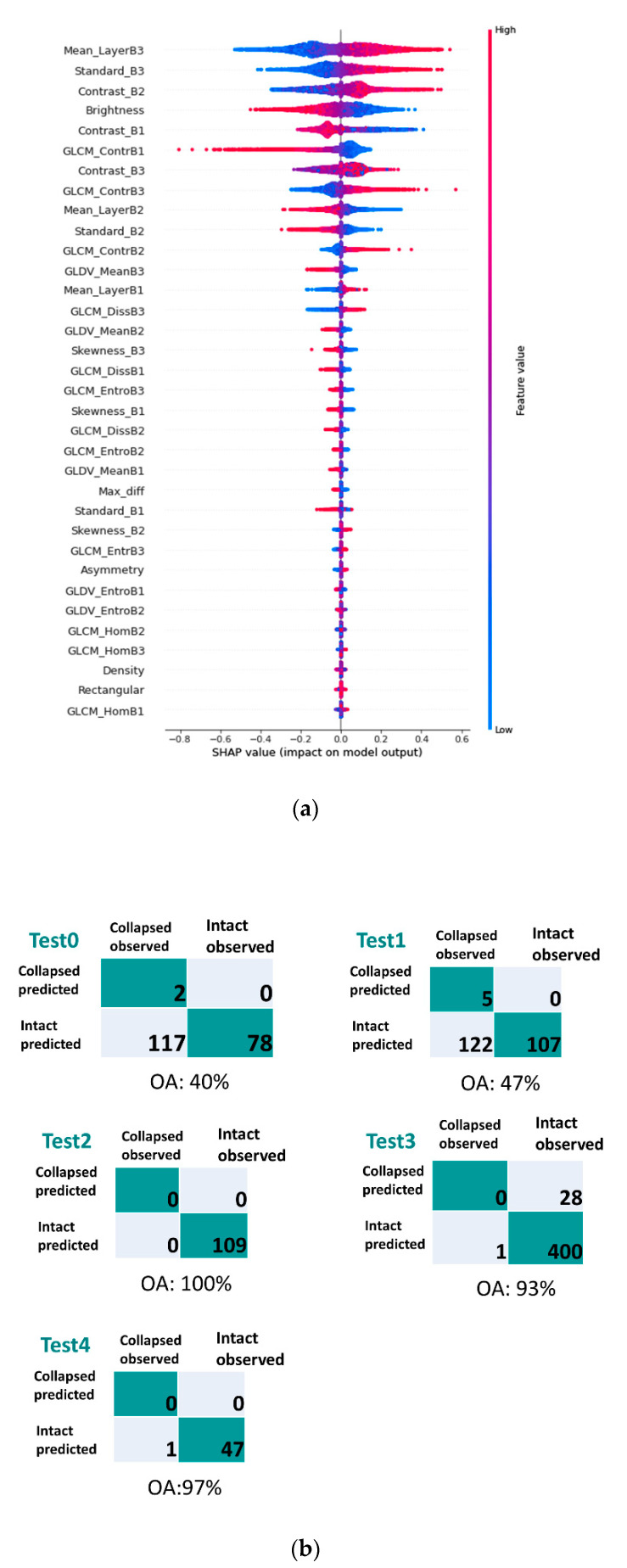
SHAP summary plot for MLP model using: (**a**) dataset A (including 15 spectral, textural, and shape features) and (**b**) the corresponding confusion matrices for different test areas.

After identifying the built-up area, 15 features, including six spectral, six textural, and three shape features (dataset A) were extracted from all of the building polygons. Spectral and textural features were calculated for each of the three bands in the image (i.e., RGB). Then the binary MLP model was trained using these features, and the SHAP values were calculated for each feature contributing to the model. Figure 5 shows the SHAP summary plot, which ranks the features based on their impact on the model output. Further, a confusion matrix has been made for each test area to measure the model performance.

The classification results show that the model performed well in detecting non-collapsed buildings. However, it failed to detect most of the collapsed buildings in all of the test areas. The reason behind a high number of true-negative results could be the fact that the dataset is skewed towards the non-collapsed buildings. The high accuracy is more evident in test 2 and test 4, as they have more non-collapsed buildings.

Regarding feature importance, as shown in the initial SHAP summary plot (Figure 5a), the spectral features have more impact on distinguishing the collapsed and non-collapsed buildings. This result is likely related to the significant difference in the roof colour of collapsed and non-collapsed buildings. In contrast, the shape features have the least impact on the model output. The low contribution of the shape features in the classification can be derived from the high similarity in the intact building shapes, which are mostly rectangles. Therefore, due to the low variance in the shape features, these features do not provide enough information to contribute to the classification, which became evident in the SHAP summary plot (Figure 5a). Moreover, the majority of the collapsed buildings occurred in the liquefaction area, where the pre-event building footprint was used to indicate the buildings as objects for analysis. Therefore, although there might be a correlation between some of the building shapes in the study area and its condition after the earthquake, generally the shape features are considered irrelevant in this study and can be removed from the model input dataset. Further, GLCM-homogeneity, GLCM-dissimilarity and GLDV-entropy are likely to be redundant due to the low contribution of these features.

**Figure 6:**
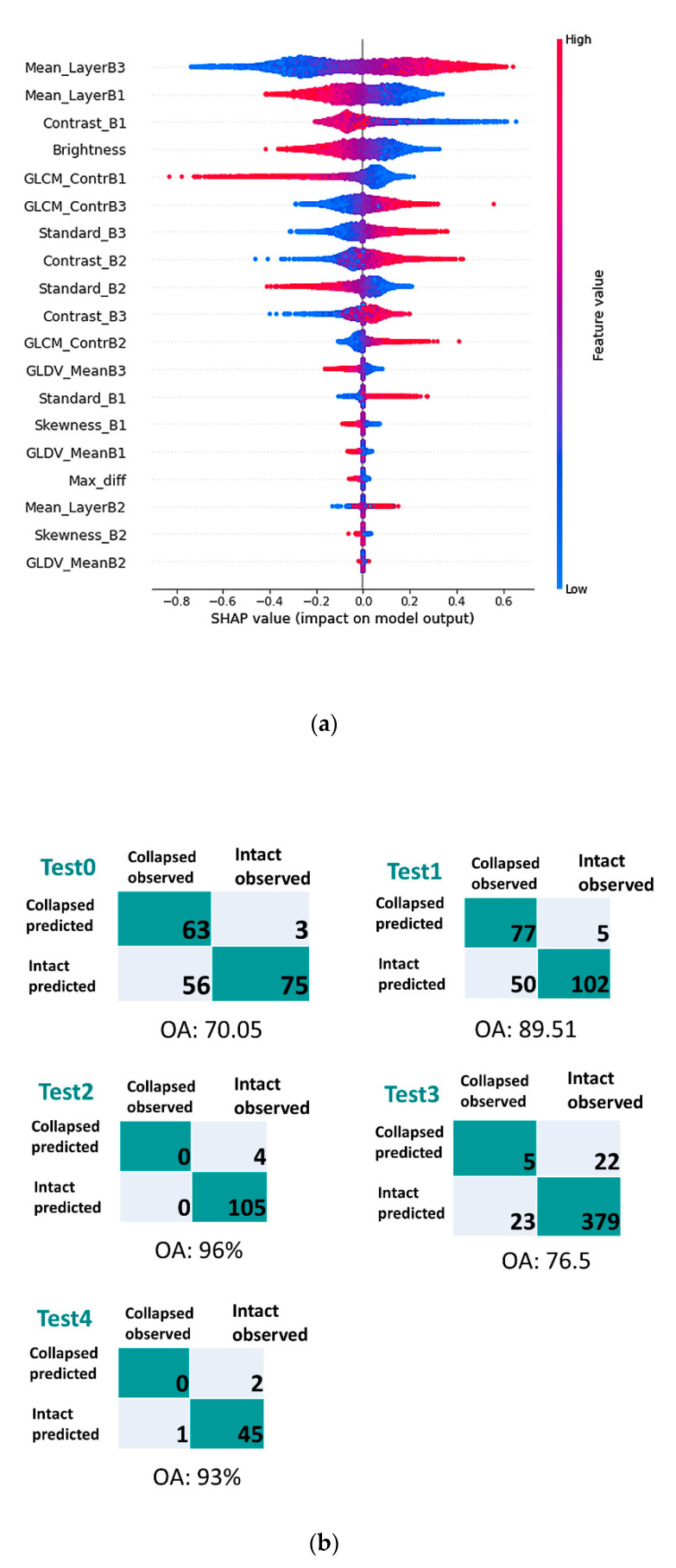
SHAP summary plot for MLP model using (**a**) dataset B (including eight spectral and textural features) (**b**) and the corresponding confusion matrices.

After removing the irrelevant and redundant features, the eight remaining spectral and textural features (dataset B) were fed into the model; and the SHAP values for all the features were calculated. Figure 6 shows the SHAP summary plot and the confusion matrices for all of the test areas using the new model. As expected, according to the new SHAP summary plot in Figure 6, the spectral features were considered the most important features for this classification. Considering the low rank of mean-layer for band 2 compared to the mean-layer for the other bands, one may argue that mean-layer is not generally an important feature. However, the variance of the green band (i.e., band 2) is significantly lower than the other bands, leading to its lower contribution to the model outcome.

The blue, purple, and red colour in the SHAP summary plot denotes the low, average, and high value of each feature for all the samples in the training dataset, respectively. The values in the *X*-axis represent the SHAP values of each observation. Therefore, the relationship between the features and the target (i.e., the building being identified as collapsed) can be explored using this plot.

As shown in Figure 6, it can be concluded that, generally, the increase in the high-rank features for band 3 (e.g., mean layer, contrast, GLCM contrast) leads to the increase in SHAP value. However, the increase in the features related to band 1 decreases the likelihood of the building to be collapsed. The reason for this might be because of the dominant red colour of the roof for most non-collapsed buildings. It is worth mentioning that the results shown above are the general feature analysis for the majority of the samples in the dataset, and the features may impact the classification output differently for individual buildings.

**Figure 7:**
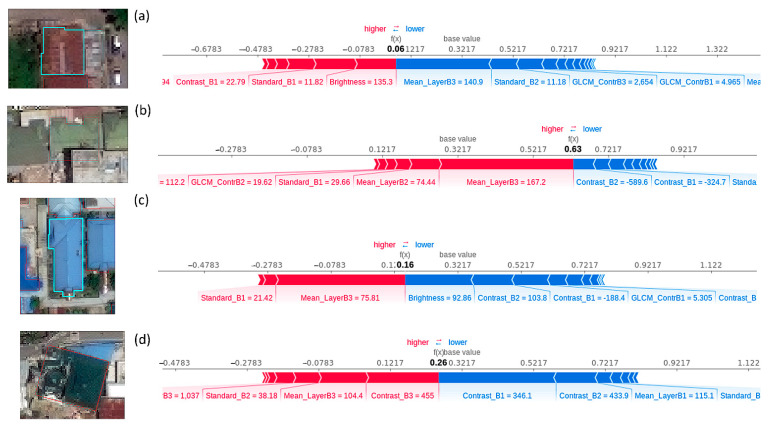
Comparison of the force plots of different buildings in the test area (**a**–**d**).

In order to locally interpret the effect of the features on the prediction, a force plot was used to investigate the impact of the features on classifying each building (Figure 7). As is shown, the MLP model could classify the buildings (a) and (c) correctly with 6% and 16% probability of the building to be collapsed, respectively.

The MLP misclassified building (b) as collapsed with 63% probability. The source of the error could be due to the angle of the image, which did not allow the building footprint to be placed correctly on the building image. Building (d) was labeled as collapsed based on the guideline in Table 3. However, it was misclassified as non-collapsed with 26% probability. As is shown (Figure 7d), half of the building roof is collapsed and the other half is completely intact, which makes classifying this building challenging. However, the model output (0.26) is very close to the base value (0.32), indicating that the impact of the features increasing the output (shown by red arrow) and the features decreasing the output (shown by blue) are almost equal. Therefore, although the result shows the building is non-collapsed, the model is not as confident as for buildings (a) and (c).

The effectiveness of the proposed explainable MLP model was compared with the random forest (RF) model. The random forest (RF) model, developed in 1990 [68], is well-known for its capability for classification and regression and is commonly used for measuring the importance of features [69]. The RF model was found to be accurate and stable in classifying high-dimension data [70]. Further, this model has been used in many building-damage-detection studies [20,71]. Therefore, in this study, the RF’s performance, along with its built-in feature importance scores, was compared with the proposed MLP model.

**Figure 8:**
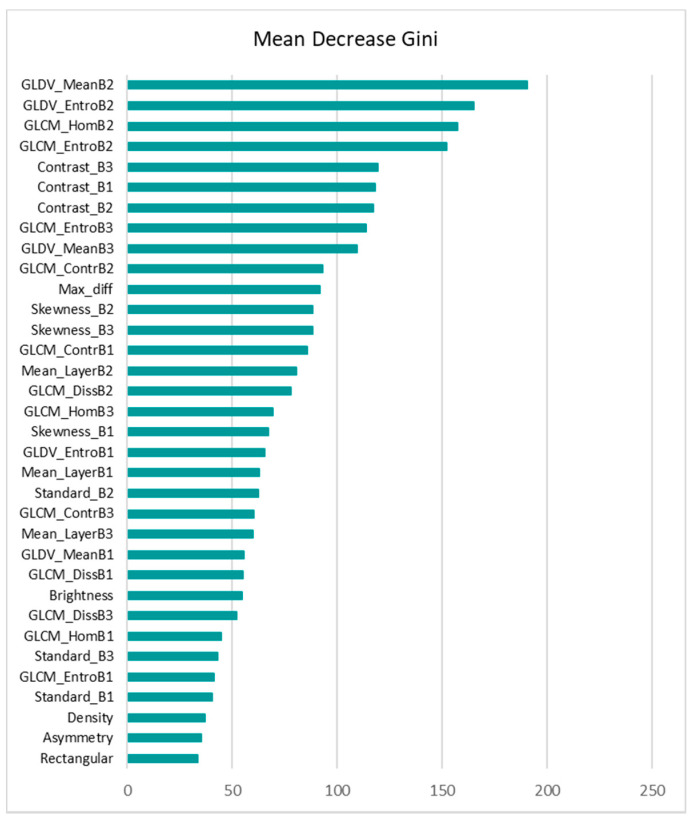
VIM plot for the RF model using 15 spectral, textural, and shape features.

Dataset A, including 15 spectral, textural and shape features, was fed into the RF model with 5000 decision trees. It is worth mentioning that different numbers of decision trees ranging from 200 to 5000 were examined, and the best result was achieved by 5000 trees. As is shown in Figure 8, the RF model ranked the shape features as the least important, which is similar to the results from the MLP SHAP summary plot. However, the most important features contributing to the RF model are quite different from that of MLP. Further, according to the RF plot, there was no significant difference between the rankings of shape features and some of the low-ranked spectral and textural features. This shows that features such as brightness were not able to contribute to the model output, as opposed to in MLP, wherein brightness was ranked as one of the important features.

**Figure 9:**
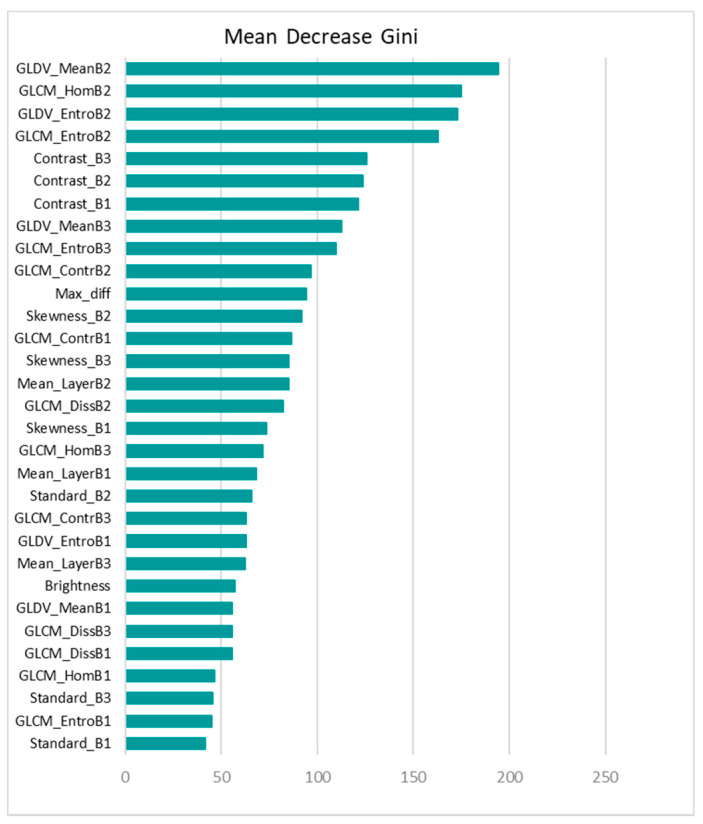
VIM plot for the RF model using eight spectral, textural features.

Regarding the performance, although both MLP and RF have similar overall accuracies, RF failed to detect almost all of the collapsed buildings. Further, using dataset B, RF did not improve the model accuracy. However, the model’s performance improved slightly after removing the three low-ranked shape features as shown in Figure 9. Both the RF ranking plot and the SHAP summary plot show the importance of the features. However, the SHAP summary plot shows the direction of the correlation (feature effect) as well.

In order to evaluate the performance of the model, the achieved results should be compared to the ground truth dataset. The building-states dataset collected through ground inspection after the earthquake is usually considered the ground truth. However, in this study, access to the ground-inspection dataset was not possible, and therefore, the Copernicus grading map was considered the ground truth. In this study, the confusion matrix was created and its parameters (i.e., overall accuracy, precision, recall, and F1 score) were calculated to quantitatively summarise the performance of the models [72]. The evaluation metrics were calculated for the MLP and RF models using 15 features (dataset A), the MLP that was built after removing some of the features (dataset B), and the RF model after removing the three low-ranked features. Table 9 summarises the overall accuracy, precision, recall, and F1 score parameters over the test area for the abovementioned models. As shown in Table 9, the MLP outperforms the RF in correctly classifying the collapsed buildings.

**Figure 10:**
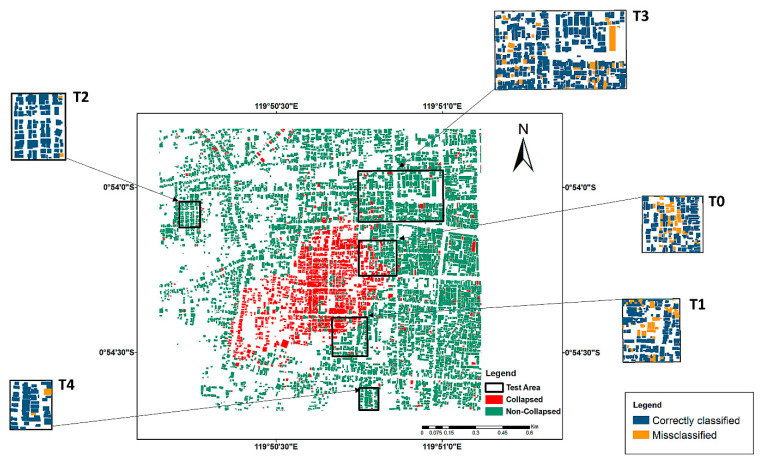
Results of building-damage mapping using all the features. The blue-orange map is the colour-coded maps of the correctly classified and misclassified buildings in all of the five test study areas.

The generated damage map is shown in Figure 10, wherein red and green indicate the classified collapsed and non-collapsed buildings, respectively. The correctly classified and misclassified buildings in the test areas are shown in blue and orange, respectively.

## 6. Conclusions

In this study, a multilayer perceptron (MLP) was employed to produce a damage map using a single post-event satellite image captured after the Palu, Indonesia earthquake in 2018. Each building was used as an object in the image using the available Open Street Map (OSM) vector data for this region. The MLP model was trained using spectral, textural, and shape features to distinguish the collapsed and non-collapsed building. Furthermore, the SHAP (Shapley additive explanation) algorithm was employed to analyse the impact of the features on the model output.

Based on the SHAP summary plot, the irrelevant and redundant features were removed from the model, and the MLP was retrained using the new set of features, resulting in the better performance of the model in detecting collapsed buildings. The SHAP force plot was utilised for some of the building samples in the dataset to analyse the effect of the features on classifying individual buildings. The performance of the model was evaluated over five diverse test areas (i.e., in terms of the number of collapsed and non-collapsed buildings within the area) using the usual evaluation metrics. The model achieved an overall accuracy of 83.68%, a precision of 80.11%, and an F1 score of 63.59%. The model performed better in the test areas where the non-collapsed buildings were predominant.

SHAP feature analysis results showed that, in general, spectral features have the most impact on detecting collapsed buildings in the study area. As discussed in Section 5, this can be a source of error when deploying the model in a geographically different study area where the spectral information of the roof images is different. Therefore, there is a need to create a training dataset that represents the variety of buildings and different collapsed types from geographical areas. As the AI industry is shifting from model-centric to data-centric, there is a necessity to focus more on the dataset being used to train the model. To this end, SHAP enables us to analyse the data in detail and guides us to build a more generic database and AI model in the future. Therefore, future work will examine the impact of the features extracted from the images covering different urban areas. The current study was a small step towards the use of explainable AI for post-earthquake building-damage mapping. Regarding the need for building a large, diverse training dataset in this domain, explainable AI can be used for detecting the limitations in training datasets that have led to a biased result.

## Figures and Tables

**Table 2:** Hyperparameters used in training the proposed MLP model in this study.

Learning Rate	0.01
Hidden Layer	2
Activation Function	ReLU
Solver	Adam
Input Nodes	50
Iterations	500

**Table 3:** Extracted GLCM and GLDV features used in this study.

Number	Feature Name	Mathematical Expression
1	GLCM Dissimilarity	$\overset{N-1}{\underset{i,j=0}{\sum }}P_{i,j}\left|i-j\right|$
2	GLCM Contrast	$\overset{N-1}{\underset{i,j=0}{\sum }}P_{i,j}{\left(i-j\right)}^{2}$
3	GLCM Homogeneity	$\overset{N-1}{\underset{i,j=0}{\sum }}\frac{P_{i,j}}{1+{\left(i-j\right)}^{2}}$
5	GLCM Entropy	$\overset{N-1}{\underset{i,j=0}{\sum }}P_{i,j}\left(-lnP_{i,j}\right)$
6	GLDV Entropy	$\overset{N-1}{\underset{K=0}{\sum }}V_{K}\left(-lnV_{K}\right)$
7	GLDV Mean	$\overset{N-1}{\underset{K=0}{\sum }}K\left(V_{K}\right)$

**Table 4:** Extracted spectral features to be used as the input features of the initial classification.

Feature Name	Mathematical Expression
Maximum Differences	$\frac{max_{i,j\epsilon K_{B}}\left|{\bar{C}}_{i}\left(v\right)-{\bar{C}}_{j}\left(v\right)\right|}{\bar{C\left(v\right)}}$
Brightness	$\frac{1}{W^{B}}\overset{K}{\underset{k-1}{\sum }}w_{k}^{B}{\bar{c}}_{k}\left(v\right)$
Mean Layer	$\frac{1}{\#P_{v}}\underset{\left(x,y,z,t\right)\epsilon P_{v}}{\sum }c_{k}\left(x,y\right)$
Standard Deviation	$\sqrt{\frac{1}{\#P_{v}}\left(\underset{\left(x,y,z,t\right)\epsilon P_{v}}{\sum }c_{k}^{2}\left(x,y\right)-\frac{1}{\#P}{\left(\underset{\left(x,y,z,t\right)\epsilon P_{v}}{\sum }c_{k}\left(x,y\right)\right)}^{2}\right)}$
Skewness	$\frac{\sum_{\left(x,y\right)\epsilon P_{v}}{\left(c_{k}\left(x,y\right)-{\bar{c}}_{k}\left(v\right)\right)}^{3}}{{\left(\sum_{\left(x,y\right)\epsilon P_{v}}{\left(c_{k}\left(x,y\right)-{\bar{c}}_{k}\left(v\right)\right)}^{2}\right)}^{\frac{3}{2}}}$
Contrast to Neighbour Pixels	1000 $\left(1-\frac{{\bar{c}}_{k\;}\left(B_{v}\left(d\right)-P_{v}\right)}{1+{\bar{c}}_{k}\left(P_{v}\right)}\right)$

**Table 5:** Shape features used in the study.

Feature Name	Mathematical Expression
Rectangular fit	$\frac{\left\{\#\left(x,y\right)\epsilon P_{v}:\;\rho_{v}\left(x,y\right)\leq 1\right\}}{\#P_{v}}$
Asymmetry	$\frac{2\sqrt{\frac{1}{4}{\left(Var\;X+Var\;Y\right)}^{2}+{\left(Var\;XY\right)}^{2}-Var\;X-Var\;Y}}{Var\;X+Var\;Y}$
Density	$\frac{\sqrt{\#P_{v}}}{1+\sqrt{Var\;X+Var\;Y}}$

**Table 6:** Overview of the source and characteristics of the datasets used in this study.

Dataset	Source	Type	Acquisition Date	Resolution
Indonesia earthquake and tsunami	DigitalGlobe	Raster (RGB)	2 October 2018	0.5 (resample)
Built-up area	OSM	Shapefile (polygon)	16 July 2019	-
EMSR317 earthquake in Indonesia	Copernicus	Shapefile (point)	30 September 2018	-

**Table 7:** Damage classification used in this study for preparing the training dataset and the number of buildings in each class.

This Study	Copernicus EMS Guideline
Non-Collapsed (Number: 6373)	No visible damage
	Possibly damaged Uncertain interpretation due to image quality Presence of possible damage proxies. Building surrounded by damaged/destroyed buildings
	Damaged Minor: The roof remains largely intact but presents partial damage Major: Partial collapse of the roof; serious failure of walls
Collapsed (Number: 2135)	Destroyed Total collapse, the collapse of part of the building (>50%); Building structure not distinguishable (the walls have been destroyed or collapsed)

**Table 8:** Overview of the quantity of collapsed and non-collapsed buildings in the training and test datasets.

Study Area	Number of Collapsed Buildings	Number of Non-Collapsed Buildings
Train	1854	5638
Test 0	119	78
Test 1	127	107
Test 2	0	109
Test 3	28	401
Test 4	1	47

**Table 9:** Overall accuracy, precision, recall, and F1 score parameters over the test area for two models.

Accuracy	Overall Accuracy (%)	Precision (%)	Recall (%)	F1 Score (%)
MLP (using dataset A)	73.55	87.5	2.54	4.94
MLP (using dataset B)	83.68	80.11	52.72	63.59
RF (using dataset A)	73.5	99	2	4
RF (after removing the three low-ranked features)	73.84	99	32.72	6.38

## Data Availability

The post-earthquake Worldview-3 satellite image was released on 2nd of October 2018 and accessed on 16 July 2019. The image can be accessed via this link: https://www.maxar.com/open-data/indonesia-earthquake-tsunami. The geospatial vector data used in this study was downloaded on 16 July 2019 from open street map. The Palu vector data can be accessed via this link: https://www.openstreetmap.org/relation/4277707#map=11/-0.7927/119.8972. The damage grading Copernicus shapefile released on 30 September 2018 for the earthquake in Indonesia (EMR317).The data was downloaded on 16 July 2019. The shapefile can be accessed via this link: https://emergency.copernicus.eu/mapping/list-of-components/EMSR317.
